# Revealing the genetic regulation of wood traits and secondary cell wall development in *Ginkgo biloba*: an integrated analysis from the perspectives of GWAS, TWAS, and WGCNA

**DOI:** 10.1093/hr/uhag062

**Published:** 2026-02-27

**Authors:** Tianhui Gao, Jiazhou Shang, Xiongjie Li, Yidong Chen, Jing Guo, Fangfang Fu, Fuliang Cao, Guibin Wang

**Affiliations:** State Key Laboratory of Tree Genetics and Breeding, Co-Innovation Center for Sustainable Forestry in Southern China, Nanjing Forestry University, Nanjing 210037, China; State Key Laboratory of Tree Genetics and Breeding, Co-Innovation Center for Sustainable Forestry in Southern China, Nanjing Forestry University, Nanjing 210037, China; State Key Laboratory of Tree Genetics and Breeding, Co-Innovation Center for Sustainable Forestry in Southern China, Nanjing Forestry University, Nanjing 210037, China; State Key Laboratory of Tree Genetics and Breeding, Co-Innovation Center for Sustainable Forestry in Southern China, Nanjing Forestry University, Nanjing 210037, China; State Key Laboratory of Tree Genetics and Breeding, Co-Innovation Center for Sustainable Forestry in Southern China, Nanjing Forestry University, Nanjing 210037, China; State Key Laboratory of Tree Genetics and Breeding, Co-Innovation Center for Sustainable Forestry in Southern China, Nanjing Forestry University, Nanjing 210037, China; State Key Laboratory of Tree Genetics and Breeding, Co-Innovation Center for Sustainable Forestry in Southern China, Nanjing Forestry University, Nanjing 210037, China; State Key Laboratory of Tree Genetics and Breeding, Co-Innovation Center for Sustainable Forestry in Southern China, Nanjing Forestry University, Nanjing 210037, China

## Abstract

Understanding the genetic and regulatory mechanisms underlying wood traits and secondary cell wall (SCW) development in *Ginkgo biloba* is crucial for improving wood quality. We identified key genes related to wood traits and SCW development through integrated genome-wide association studies (GWAS), transcriptome-wide association studies (TWAS), and weighted gene co-expression network analysis (WGCNA). Cellulose biosynthesis in the SCW is catalyzed by the CesA4–CesA7–CesA8 complex encoded by *GbCesA4*, *GbCesA7*, and *GbCesA8A*/*8B*. These *CesA* genes form a co-expression network with *TUBA*/*TUBB* and *EG*, indicating coordination among cellulose synthesis, cytoskeletal guidance, and cell wall remodeling. Additionally, loss of function of *GbCesA8B* caused only a slight reduction in cellulose content, supporting potential functional redundancy between *GbCesA8A* and *GbCesA8B*. For hemicellulose biosynthesis, *GbCSLA9A*/*9B* and *IRX9*/*IRX14* were major contributors to mannan/glucomannan and xylan synthesis, respectively, and formed a co-expression network with *UXS*, *UXE*, *IRX7*, *GXMT*, and *URGT*, spanning nucleotide sugar supply, transport, and polymer elongation and modification. Moreover, *MYB46* may regulate mannan/glucomannan biosynthesis in the SCW by activating *CSLA9* transcription. For lignin biosynthesis, TWAS identified multiple genes involved in phenylalanine biosynthesis, phenylpropanoid metabolism, and lignin monomer polymerization, including *ADT*/*PDT*, *PAL*, and *PER*, as well as *MYB91* and several *bHLH* genes that may positively regulate lignin accumulation. Furthermore, several transcription factors potentially involved in SCW development were identified, including *GATA9* as a putative positive regulator, *WRKY12* and *HB15* as potential negative regulators, and *ELF6*, which may facilitate tracheid expansion. Our findings provide valuable insights into the genetic regulation of wood traits and SCW development in *Ginkgo*.

## Introduction

Wood, a crucial renewable resource, plays a pivotal role in human production and daily life. The characteristics of wood determine its physical and mechanical properties, processability, durability, and potential applications across various domains. During the secondary growth phase of trees, the proliferation and specialization of cambium cells govern the formation of wood structure, while the biogenesis and assembly of the secondary cell wall (SCW) directly influence the physical and chemical properties of wood. Therefore, comprehensively elucidating the genetic foundations of wood traits and uncovering the molecular mechanisms regulating SCW development are fundamental prerequisites for improving wood quality, enhancing processing efficiency, and promoting the molecular breeding of superior timber species.

In recent years, significant progress has been made in understanding the molecular mechanisms governing wood formation and SCW development in both model plants and commercial tree species. The differentiation of xylem mother cells and the subsequent development of secondary xylem are precisely regulated by a multilayered signaling network. In *Arabidopsis thalian*a, a relatively comprehensive multilayered regulatory network involving NAC, MYB, and other transcription factors has been established, revealing the core regulatory framework for SCW formation [1]. Similarly, in the model tree *Populus*, numerous structural and transcription factor genes such as *CesA* [2], *CSLA* [3], *MYB10* [4], *MYB031* [5], *MYB90* [6], *MYB92* [7], *MYB120* [8], *WRKY15* [9], and *bHLH125* [10] have been identified as being involved in SCW development, offering valuable insights into the regulatory mechanisms governing xylem development.

*Ginkgo biloba*, the oldest living gymnosperm, provides a unique perspective for understanding the evolutionary history and genetic basis of wood formation that is absent from studies of current commercial timber species. In addition, *Ginkgo* wood also exhibits excellent material properties and high market value, giving it substantial commercial potential in the future. However, compared with conifers and most angiosperms [11–13], our understanding of the genetic mechanisms underlying wood formation in *Ginkgo*, particularly secondary xylem development, remains limited. The functional genes and regulatory networks related to this process have yet to be systematically elucidated [14, 15]. In our previous investigation of the seasonal development of *Ginkgo* xylem, we identified and constructed multiple non-coding RNA regulatory modules that modulate SCW development by regulating hormone signaling pathways and the expression of relevant regulatory genes [16], thereby enriching the regulatory network of SCW development in ginkgo to some extent.

Since its initial proposal in 1996 and subsequent validation in age-related retinal macular degeneration research in 2005 [17, 18], genome-wide association studies (GWAS) have emerged as a pivotal tool for elucidating the genetic mechanism of complex traits. Driven by continuous advancements in sequencing technologies and the rapid evolution of bioinformatic tools, GWAS have been extensively applied not only in medicine but also in major crops such as *Oryza sativa* [19], *Gossypium* [20, 21], and *Triticum aestivum* [22], as well as in studies of forest tree traits. In tree species such as *Populus trichocarpa* [23], *Populus deltoides* [24], *Picea glauca* [25], *Eucalyptus globulus* [26], and *Cryptomeria japonica* [27], numerous functional gene loci associated with wood quality and yield have been identified through GWAS. However, a major challenge is that most significant variants detected by GWAS are located in non-coding regions, which complicates the direct identification of causal genes responsible for trait variation.

In contrast, transcriptome-wide association studies (TWAS) enable the direct association between gene expression and traits, thereby facilitating more effective identification of potential functional genes. Since their proposal in 2016 [28], TWAS have been widely used to investigate various traits in animals and plants. For example, Tang et al. [29] employed TWAS to identify hundreds of genes, including several NAC, WRKY, and AP2/ERF family transcription factors, that were significantly associated with oil content in *Brassica napus*. Similarly, Li et al. [30] integrated GWAS and TWAS to uncover key genes involved in cotton fiber development and SCW biosynthesis. Notably, although GWAS has been applied to study seed traits and secondary metabolism in *G. biloba* [31, 32], investigations of wood properties in this species using GWAS or TWAS have not yet been reported.

In this study, 290 *Ginkgo* accessions with abundant genetic diversity were used as experimental materials. Based on whole-genome resequencing data and transcriptome sequencing data obtained during key stages of xylem development, we systematically identified key genes governing variation in *Ginkgo* wood traits by integrating GWAS, TWAS, and weighted gene co-expression network analysis (WGCNA). We particularly focused on the genetic regulation of the biosynthesis of major SCW components (lignin, cellulose, and hemicellulose) to identify structural genes, transcription factors, and potential synergistic regulatory mechanisms involved in their biosynthetic processes. This work aims to elucidate the genetic mechanisms underlying wood formation and SCW development and to provide a theoretical foundation and genetic resources for the molecular breeding of superior *Ginkgo* wood.

## Results

### The pattern of SNP and InDel variations across the *G. biloba* genome

**Figure 1:**
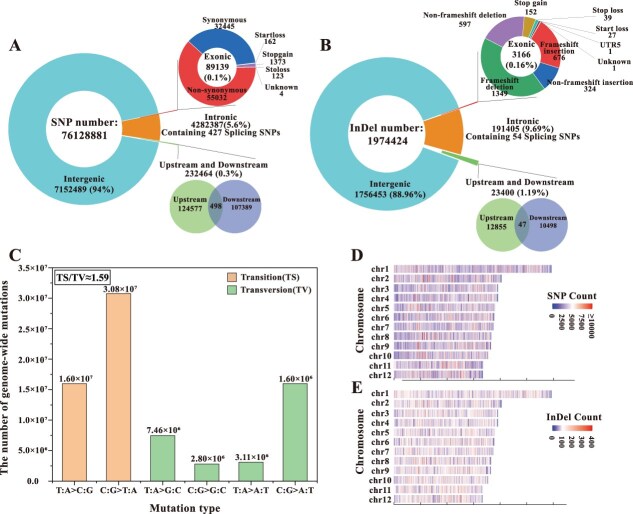
The pattern of genomic variation in ginkgo. (A, B) Annotation results of SNPs and InDels in the ginkgo genome. (C) Distribution of SNP mutation types in the ginkgo genome. (D, E) Density of SNPs and InDels on chromosomes in the ginkgo genome.

This study performed whole-genome resequencing of 290 *G. biloba* samples at an average depth of 7.6×, with high sequencing quality (Q20 > 98%, Q30 > 94%) and normal GC content (34%–38%) (Table S3). After quality control, clean reads were aligned to the reference genome using BWA software, resulting in an average mapping rate of 99.34% (Table S3). Subsequently, variant detection was performed using GATK 4.5. Following a stringent filtering process, a total of 76 128 881 high-quality SNPs and 1 974 424 InDels were identified (Fig. 1A and B).

The distribution of SNPs and InDels across the ginkgo genome was relatively uniform (Fig. 1D and E). Additionally, several chromosomes exhibited multiple regions with high SNP or InDel densities (Fig. 1D and E). Analysis of mutation types revealed a higher frequency of transition mutations (T:A > C:G and C:G > T:A) than transversion mutations (T:A > G:C, C:G > G:C, T:A > A:T, and C:G > A:T), with a transition-to-transversion ratio (TS/TV) of ~1.59 (Fig. 1C). Notably, C:G > T:A transitions were the most prevalent mutation type, far exceeding other categories (Fig. 1C).

Annotation results showed that the majority of SNPs (94%) and InDels (88.96%) were located in intergenic regions (Fig. 1A and B). Among the 89 139 SNPs identified in exonic regions, 55 032 (61.74%) were non-synonymous SNPs, potentially affecting protein function by altering amino acids (Fig. 1A). Furthermore, among the 3166 exonic InDels, 2025 (63.96%) were frameshift InDels, while 921 (29.09%) were non-frameshift InDels (Fig. 1B). In addition, 1658 SNPs and 218 InDels were identified that caused gain or loss of translation start/stop sites, resulting in failed protein translation, premature termination, or extended translation (Fig. 1A and B). Such variants are particularly deleterious as they often result in complete loss of protein function.

### Genome-wide association study for 12 wood traits

The establishment of a high-density variation map of the ginkgo genome has provided a wealth of precise and accurate genetic markers for conducting GWAS. To pinpoint key genes associated with variation in ginkgo wood traits, we performed GWAS using GEMMA and further referred to GWAS results obtained with EMMAX to enhance the reliability of associated sites. The results revealed a high degree of consistency between the two software packages (Table S4, Figs S1–S6). Except for an abnormal increase in InDel association results detected by EMMAX for some traits, such as tracheid length to width ratio (TLW), tracheid width to lumen ratio (TWL), and tracheid wall thickness (TWT), the vast majority of loci were consistently identified by both software packages. Because the association results from GEMMA were more accurate and reliable than those from EMMAX, we primarily focused on the GEMMA results for subsequent analyses.

Using GEMMA, a total of 1650 SNPs and 196 InDels associated with ginkgo wood traits were identified (Table S4.1). Among the various traits examined, cellulose content (CC) displayed the highest number of associated signals, with 1343 SNPs (81.39%) and 56 InDels (28.57%), followed by TWL and cellulose crystallinity degree (CCD), which exhibited 98 (73 SNPs and 25 InDels) and 94 (68 SNPs and 26 InDels) variant sites, respectively (Table S4.1). Furthermore, four pleiotropic SNPs (chr10:362509447, chr10:365643063, chr10:365709595, chr10:365902016) and three pleiotropic InDels (chr10:367769028, chr10:36855634, chr10:370533958) exhibited dual-trait associations with wood basic density (WBD) and wood oven-dry density (WOD) (Table S4.1). Similarly, two pleiotropic SNPs (chr2:532772394 and chr12:279586611) were identified for TWL and TWT (Table S4.1).

Results from EMMAX also showed that cellulose content was the trait with the most associated signals, with a total of 1282 SNPs and 54 InDels detected, all of which overlapped with associations identified by GEMMA for cellulose content (Table S4, Figs S5 and S6). Based on the principle of linkage disequilibrium, we delineated association regions by extending 100 kb upstream and downstream of associated sites, identifying 180 linked genes (Table S5). Among these, 120 genes harbored non-synonymous SNPs or frameshift/non-frameshift InDels in exonic regions, while 167 genes contained SNPs or InDels in upstream regions, resulting in a total of 172 genes with such variations (Table S5).

Using published RNA-seq data [16], we further analyzed the expression patterns of these 172 genes to narrow the range of candidate genes. After excluding genes with low expression levels (mean FPKM < 0.5), 116 candidate genes were retained for further investigation (Table S5). For wood oven-dry density, 15 candidate genes were identified (Table S5). Among them, *evm.TU.chr3.1073* (*TAA1*) encodes an L-tryptophan-pyruvate aminotransferase, catalyzing the transfer of an amino group from L-tryptophan to pyruvate. Additionally, *evm.TU.chr10.1203* (*XYL4*), encoding xylan 1,4-β-xylosidase, and *evm.TU.chr7.1369* (*MAN*), encoding mannan endo-1,4-β-mannosidase, were identified as potential genes associated with WBD (Table S5).

Among the 48 candidate genes related to tracheid morphological traits including tracheid length (TL), tracheid width (TW), TLW, TWT, and TWL, several functional genes or transcription factors with regulatory potential were identified, including *evm.TU.chr6.1080* (*EIN3*) and *evm.TU.chr6.1069* (*ABI5*). Furthermore, *evm.TU.chr1.2638* (*CCR*), encoding cinnamoyl-CoA reductase, a key enzyme in lignin precursor conversion crucial for SCW formation, was identified (Table S5). These genes serve as potential candidates for tracheid morphogenesis and regulation.

### Identification of key genes for cellulose synthesis

**Figure 2:**
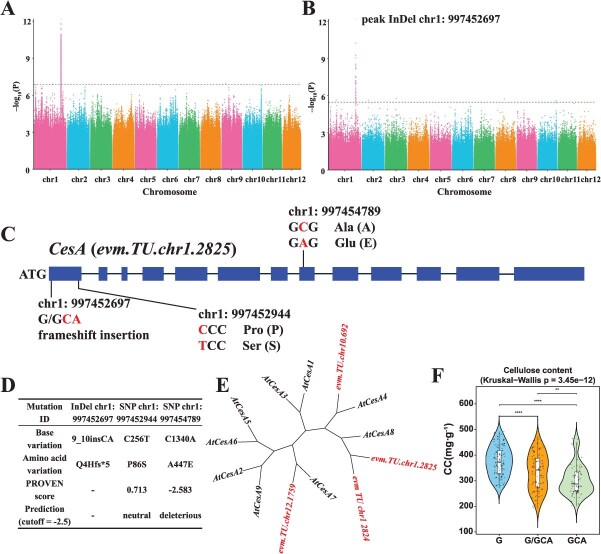
Functional characterization of *evm.TU.chr1.2825*. (A) Manhattan plot of GWAS results based on SNPs and cellulose content. (B) Manhattan plot of GWAS results based on InDels and cellulose content. (C) Gene structure of *evm.TU.chr1.2825*. Blue rectangles and black lines indicate exons and introns, respectively. (D) Effects of mutation sites within *evm.TU.chr1.2825* on protein function. A PROVEAN score below −2.5 is considered deleterious, whereas scores above this threshold are regarded as neutral. (E) Phylogenetic analysis of *CesA* family genes in *G. biloba* and *A. thaliana*. (F) Violin plot of cellulose content based on the genotype of *evm.TU.chr1.2825*. Statistical analysis was performed using the Kruskal−Wallis test; *, **, ***, and **** represent *P* < 0.05, *P* < 0.01, *P* < 0.001, and *P* < 0.0001, respectively.

Cellulose, the predominant structural polysaccharide in plant cell walls, is essential for providing structural support and protection to plant cells. As mentioned above, cellulose content is the trait with the highest number of associated signals (Table S4.1). Notably, a prominent enrichment of association signals was observed within the range of 997 196 864 to 998 048 937 bp on chromosome 1 (chr1), suggesting that this region may contain critical genetic loci for cellulose biosynthesis in ginkgo. Within this region, a potential candidate gene, *evm.TU.chr1.2825*, was pinpointed and annotated as a cellulose synthase (*CesA*) gene (Table S5). Phylogenetic analysis revealed a high degree of homology between *evm.TU.chr1.2825* and *AtCesA8*, implying functional similarity or evolutionary conservation between the two genes (Fig. 2E).

Mutation annotation revealed that the peak InDel (chr1:997452697) associated with the cellulose content was a frameshift insertion mutation (Fig. 2B and C). This mutation inserts ‘CA’ after the ninth base of the first exon of *evm.TU.chr1.2825* and leads to premature termination of translation at the fifth amino acid after insertion, resulting in complete loss of gene function (Fig. 2D). Furthermore, two non-synonymous SNPs were observed in this gene, causing amino acid substitutions at positions 86 and 447 (Fig. 2C). According to PROVEAN predictions, these two mutations were evaluated as neutral (0.713) and deleterious (−2.583), respectively (Fig. 2D). Therefore, it is inferred that the mutation at amino acid position 86 does not significantly affect protein function, whereas the mutation at position 447 may have a substantial impact. Genotyping based on the InDel chr1:997452697 showed that samples carrying the InDel had significantly lower cellulose content than those without it (Fig. 2F).

### Identification of key genes for hemicellulose synthesis

**Figure 3:**
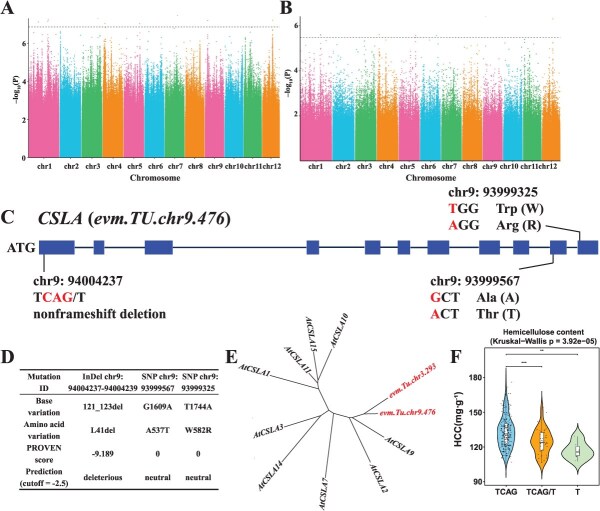
Functional characterization of *evm.TU.chr9.476*. (A) Manhattan plot of GWAS results based on SNPs and hemicellulose content. (B) Manhattan plot of GWAS results based on InDels and hemicellulose content. (C) Gene structure of *evm.TU.chr9.476*. Blue rectangles and black lines indicate exons and introns, respectively. (D) Effects of mutation sites within *evm.TU.chr9.476* on protein function. A PROVEAN score below −2.5 is considered deleterious, whereas scores above this threshold are regarded as neutral. (E) Phylogenetic analysis of *CSLA* family genes in *G. biloba* and *A. thaliana*. (F) Violin plot of hemicellulose content based on the genotype of *evm.TU.chr9.476*. Statistical analysis was performed using the Kruskal–Wallis test; *, **, ***, and **** represent *P* < .05, *P* < .01, *P* < .001, and *P* < .0001, respectively.

Hemicellulose, an essential component of the plant cell wall, enhances strength and tensile resistance by interacting with cellulose through hydrogen and covalent bonds. In the GWAS of hemicellulose content (HCC), we identified a total of 15 variant sites and screened out five candidate genes (Tables S4 and S5). One of the candidate genes, *evm.TU.chr9.476* located on chromosome 9 (chr9), was annotated as encoding β-mannan synthase (CSLA), an enzyme responsible for elongating β-1,4-mannan chains using GDP-Man as a substrate to produce mannan backbones in hemicellulose (Table S5). Phylogenetic analysis indicated that this gene shares homology with *AtCSLA9* in *A. thaliana* (Fig. 3E).

Further analysis revealed the presence of one non-frameshift deletion (chr9:94004237) and two non-synonymous SNPs (chr9:93999325 and chr9:93999567) in the exons of *evm.TU.chr9.476* (Fig. 3C). Specifically, the non-frameshift deletion resulted in the loss of leucine at position 41, while the two non-synonymous SNPs led to the substitution of tryptophan (W) with arginine (R) at amino acid position 582 and alanine (A) with threonine (T) at position 537, respectively (Fig. 3C). Functional domain analysis using InterPro revealed that leucine at position 41 resides within the transmembrane helix region (TMhelix), as predicted by TMHMM, whereas tryptophan at position 582 and alanine at position 537 are situated in the cytoplasmic domain predicted by Phobius. Further evaluation of the variants using PROVEAN revealed that only the deletion of leucine at position 41 was predicted to be deleterious (−9.189) (Fig. 3D).

Therefore, we conducted genotyping based on the InDel chr9:94004237. The results showed that hemicellulose content was significantly higher in individuals with the homozygous TCAG genotype (retaining leucine at position 41) than in those with the heterozygous deletion genotype (TCAG/T) and the homozygous deletion genotype (T) (Fig. 3F). Moreover, individuals with the T genotype exhibited lower hemicellulose content than those with the TCAG/T genotype (Fig. 3F).

### Transcriptome-wide association study for 12 wood traits

In this study, after eliminating low-expression genes (mean FPKM < 0.5) from 290 samples, we retained 18 203 genes and conducted TWAS using the linear mixed model (LMM) implemented in the GEMMA software (Table S6, Fig. S7). In the TWAS of 12 wood traits, no significant (FDR < 0.01) association genes were identified for microfibril angle (MFA), whereas an excessively large number of significant association genes were detected for CCD, TW, and TWT—specifically, 8638, 6739, and 1931 genes, respectively (Table S6, Fig. S7). Therefore, further in-depth analysis was not pursued for these traits. For the remaining eight wood traits, a total of 2333 genes exhibited significant associations (Table S6, Fig. S7). Notably, lignin content displayed the highest number of significantly associated genes (1335), followed by tracheid length (471 genes) (Table S6, Fig. S7).

**Figure 4:**
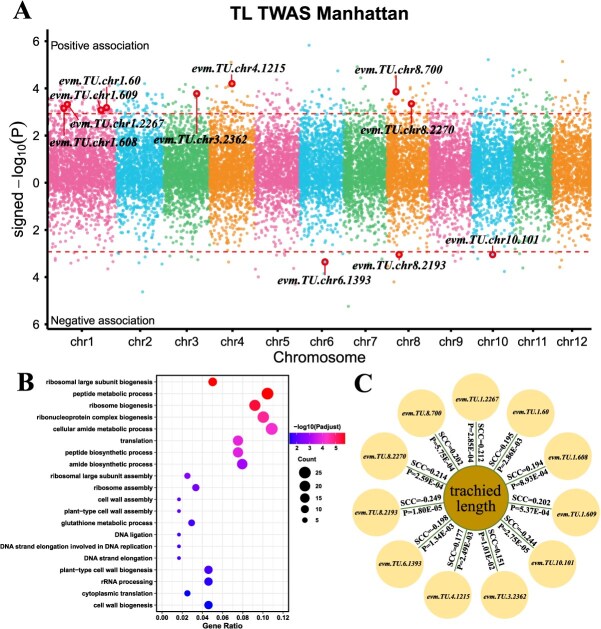
TWAS of tracheid length. (A) Manhattan plot of TWAS based on tracheid length. Some genes significantly associated with tracheid length are marked in the plot. (B) GO enrichment of genes significantly associated with tracheid length in TWAS. (C) Correlation analysis of genes enriched in plant-type cell wall biogenesis (GO:0009832) and plant-type cell wall assembly (GO:0071668) with tracheid length. SCC indicates the Spearman correlation coefficient.

GO enrichment analysis of genes significantly associated with tracheid length revealed that, apart from terms related to ribosome formation and translation, the remaining genes were predominantly enriched in processes pertaining to cell wall development, including plant-type cell wall biogenesis (GO:0009832) and plant-type cell wall assembly (GO:0071668) (Fig. 4B). This indicates that these associated genes may be related to the synthesis and assembly of the cell wall. Since the development and structural formation of tracheids depend on the deposition and ordered assembly of SCW components, we further propose that these associated genes may be involved in wood tracheids formation. Among the eleven genes enriched in these two terms, eight were significantly positively correlated with tracheid length, whereas three were negatively correlated with tracheid length (Fig. 4A and C). However, these genes lack detailed functional annotations; therefore, their specific roles in cell wall deposition and assembly in ginkgo require further investigation.

**Figure 5:**
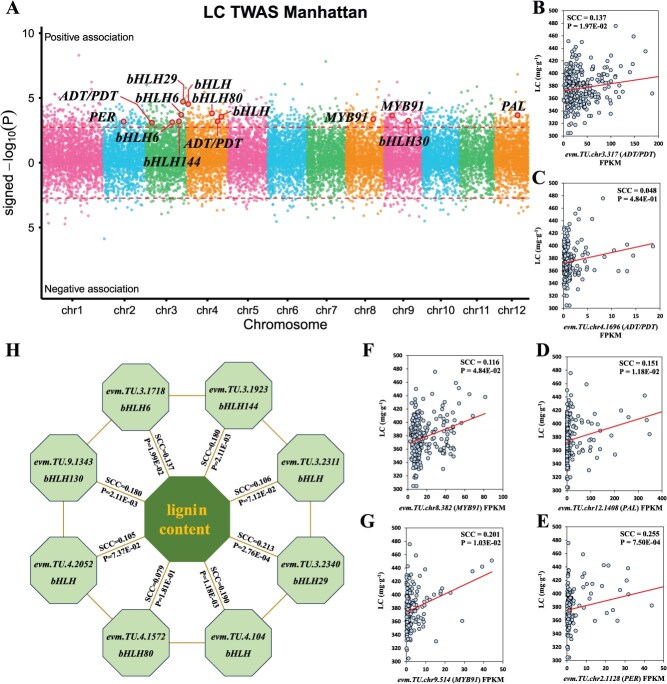
TWAS of lignin content. (A) Manhattan plot of TWAS based on lignin content. Some genes significantly associated with lignin content are marked in the plot. (B–G) Correlation analysis of *evm.TU.chr3.317* (*ADT/PDT*), *evm.TU.chr4.1696* (*ADT/PDT*), *evm.TU.chr12.1408* (*PAL*), *evm.TU.chr2.1128* (*PER*), *evm.TU.chr8.382* (*MYB91*), and *evm.TU.chr9.514* (*MYB91*) with lignin content. (H) Correlation analysis of eight *bHLH* genes with lignin content.

We identified multiple structural and transcription factor genes that were significantly positively associated with lignin content through TWAS, including *evm.TU.chr3.317* and *evm.TU.chr4.1696*, encoding arogenate/prephenate dehydratase (ADT/PDT); *evm.TU.chr12.1408* encoding phenylalanine ammonia-lyase (PAL); *evm.TU.chr2.1128* encoding peroxidase (PER); and *evm.TU.chr8.382* and *evm.TU.chr9.514*, encoding MYB91 (Fig. 5A–G). In addition, among the significantly associated genes, all members of the *bHLH* family showed a significant positive correlation with lignin content, including *evm.TU.chr3.1718*, *evm.TU.chr3.1923*, *evm.TU.chr3.2311*, *evm.TU.chr3.2340*, *evm.TU.chr4.104*, *evm.TU.chr4.1572*, *evm.TU.chr4.2052*, and *evm.TU.chr9.1343* (Fig. 5H).

**Figure 6:**
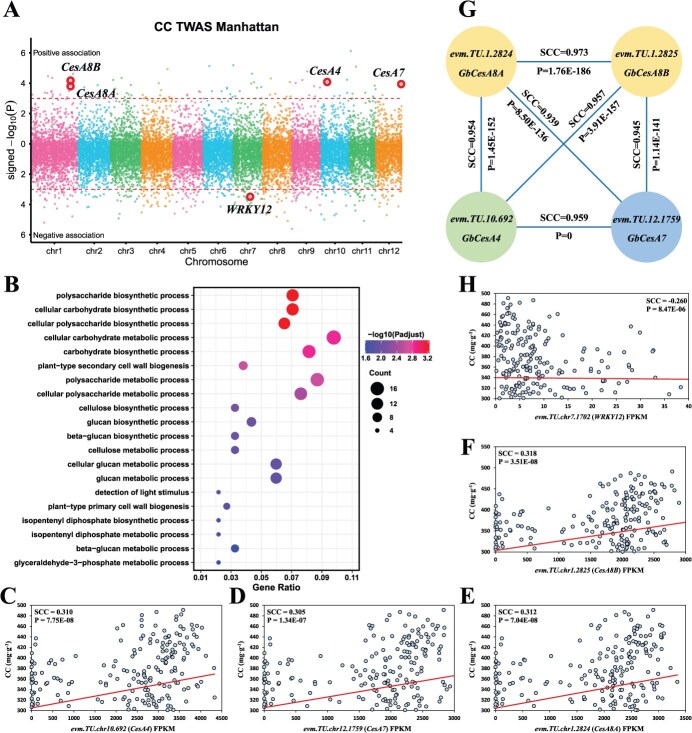
TWAS of cellulose content. (A) Manhattan plot of TWAS based on cellulose content. Some genes significantly associated with cellulose content are marked in the plot. (B) GO enrichment of genes significantly associated with cellulose content in TWAS. (C–F) Correlation analysis of *evm.TU.chr10.692* (*GbCesA4*), *evm.TU.chr12.1759* (*GbCesA7*), *evm.TU.chr1.2824* (*GbCesA8A*), and *evm.TU.chr1.2825* (*GbCesA8B*) with cellulose content. (G) Correlation analysis among the four *CesA* genes. (H) Correlation analysis of *evm.TU.chr7.1702* (*WRKY12*) with cellulose content.

For cellulose content, a total of 363 genes significantly associated with this trait were identified (Fig. 6A). GO enrichment analysis of these genes revealed predominant enrichment in processes related to polysaccharide and carbohydrate metabolism and biosynthesis (Fig. 6B). Significant enrichment was also observed for terms such as cellulose biosynthetic process (GO:0030244), cellulose metabolic process (GO:0030243), and plant-type secondary cell wall biogenesis (GO:0009834) (Fig. 6B). These findings emphasize that the identified genes may be involved in cellulose biosynthesis and SCW development. Among the genes enriched in the cellulose biosynthetic process (GO:0030244), four of six were annotated as cellulose synthase (*CesA*) genes and displayed significant positive correlations with cellulose content (Fig. 6A, C–F). Phylogenetic analysis of these genes alongside *CesA* genes in *A. thaliana* revealed that *evm.TU.chr1.2824* and *evm.TU.chr1.2825* were homologous to *AtCesA8* and were thus designated as *GbCesA8A* and *GbCesA8B*, respectively (Fig. 2E). Similarly, *evm.TU.chr10.692* and *evm.TU.chr12.1759* were homologous to *AtCesA4* and *AtCesA7*, respectively, and were named *GbCesA4* and *GbCesA7* (Fig. 2E). All four *CesA* genes exhibited high expression levels in the xylem of *G. biloba*, and showed highly correlated expression patterns (Fig. 6G). Additionally, we identified a gene, *evm.TU.chr7.1702*, encoding WRKY12, which showed a negative correlation with cellulose content (Fig. 6H).

**Figure 7:**
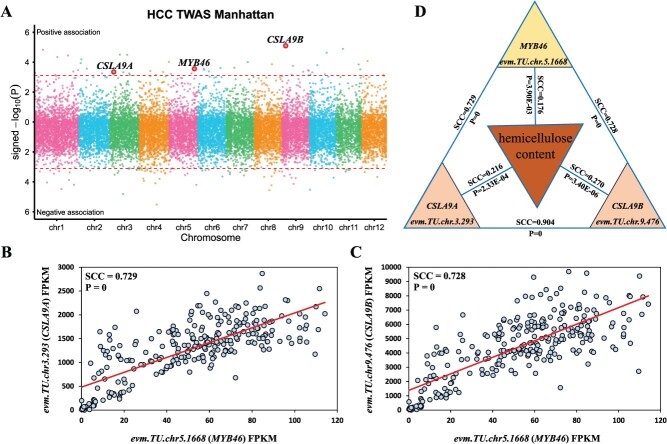
TWAS of hemicellulose content. (A) Manhattan plot of TWAS based on hemicellulose content. Some genes significantly associated with hemicellulose content are marked in the plot. (B, C) Correlation analysis of *evm.TU.chr5.1668* (*MYB46*) with *evm.TU.chr3.293* (*GbCSLA9A*) and *evm.TU.chr9.476* (*GbCSLA9B*). (D) Correlation analysis of *evm.TU.chr5.1668* (*MYB46*), *evm.TU.chr3.293* (*GbCSLA9A*), and *evm.TU.chr9.476* (*GbCSLA9B*) with hemicellulose content.

Among the 139 genes significantly associated with hemicellulose content, two genes, *evm.TU.chr3.293* and *evm.TU.chr9.476*—located on different chromosomes, were identified; both encode beta-mannan synthase (CSLA), an enzyme responsible for mannan biosynthesis (Fig. 7A). Expression analysis revealed strong positive correlations between these genes and hemicellulose content (Fig. 7D). Phylogenetic analysis further indicated that both genes are homologous to *AtCSLA9* in *A. thaliana*, leading to their designation as *GbCSLA9A* and *GbCSLA9B*, respectively (Fig. 3E). In addition, a transcription factor gene, *evm.TU.chr5.1668* (*MYB46*), exhibited significant positive correlations with hemicellulose content, as well as with *GbCSLA9A* and *GbCSLA9B* (Fig. 7B–D).

### Weighted gene co-expression network analysis and identification of the hub genes

**Figure 8:**
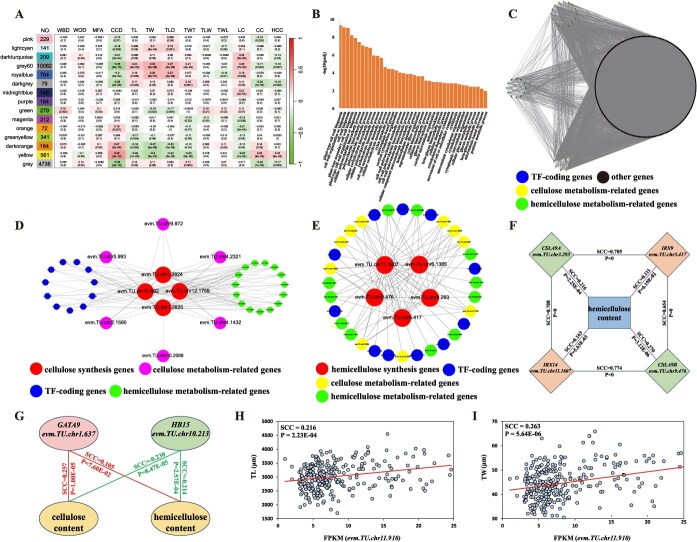
WGCNA and identification of hub genes in SCW development. (A) Correlations between module eigengenes and wood traits. Each cell contains the correlation coefficient and *P*-value between the module and the trait. (B, C) GO enrichment analysis (B) and co-expression network (C) of genes with TOM > 0.1 in the yellow module. (D) The co-expression network centered on *CesA* genes. (E) The co-expression network centered on *CSLA* and *IRX* genes. (F) Correlation analysis among *evm.TU.chr3.293* (*GbCSLA9A*), *evm.TU.chr9.476* (*GbCSLA9B*), *evm.TU.chr5.417* (*IRX9*), *evm.TU.chr11.1607* (*IRX14*), and hemicellulose content. (G) Correlation analysis of *evm.TU.chr1.637* (*GATA9*) and *evm.TU.chr10.213* (*HB15*) with cellulose and hemicellulose content. (H, I) Correlation analysis of *evm.TU.chr11.910* (*ELF6*) with tracheid length and tracheid width.

Through WGCNA, we identified a total of 14 gene expression modules encompassing 13 465 genes (Fig. 8A). An additional 4738 genes were unassigned to any of the identified modules and were therefore classified into the gray module (unclassified module) (Fig. 8A). Among the 14 modules, the darkturquoise (*r* = 0.24, *P* = 5e−05), grey60 (*r* = 0.23, *P* = 7e−05), and midnightblue (*r* = 0.21, *P* = 3e−04) modules exhibited significant positive correlations with lignin content, implying that these three modules are, to some extent, related to the process of lignin synthesis and accumulation (Fig. 8A). Furthermore, the grey60, royalblue, and darkgrey modules showed significant correlations with various tracheid morphological traits, such as tracheid length (TL), tracheid width (TW), and tracheid wall thickness (TWT), suggesting that they may have potential links with tracheid development (Fig. 8A). Notably, the yellow module displayed significant positive correlations with cellulose content (*r* = 0.23, *P* = 9e−05), hemicellulose content (*r* = 0.19, *P* = 9e−04), and cellulose crystallinity degree (*r* = 0.39, *P* = 3e−12), indicating that this module may be closely related to the regulation of SCW formation and wood structural characteristics in ginkgo (Fig. 8A).

To further elucidate the biological functions of genes within the yellow module, we selected genes with TOM > 0.1 for construction of a co-expression network and GO enrichment analysis (Fig. 8B and C). The analysis revealed significant enrichment of terms closely associated with SCW biosynthesis and related processes, including the synthesis and metabolism of cellulose (GO:0030244, GO:0030243), hemicellulose (GO:0010410), β-glucan (GO:0051274, GO:0051273), xylan (GO:0045491, GO:0045492), cell wall polysaccharide (GO:0010383, GO:0070592), and cell wall macromolecule (GO:0044036, GO:0044038) (Fig. 8B). These results indicate that the yellow module may play a significant role in the synthesis and remodeling of SCW components in ginkgo.

Notably, the co-expression network highlighted four hub genes with the highest connectivity, namely *evm.TU.chr10.692* (*GbCesA4*), *evm.TU.chr12.1759* (*GbCesA7*), *evm.TU.chr1.2825* (*GbCesA8A*), and *evm.TU.chr1.2824* (*GbCesA8B*) (Fig. 8D). All of these genes encode cellulose synthase (CesA) and are fully consistent with the four *CesA* genes identified by TWAS (Fig. 6A). In addition, several genes closely related to cellulose synthesis or remodeling were also identified in the network, including *TUBA* (*evm.TU.chr2.1590*), encoding α-tubulin; *TUBB* (*evm.TU.chr9.972*, *evm.TU.chr10.2089*, and *evm.TU.chr4.2321*), encoding β-tubulin; and *EG* (*evm.TU.chr4.1432* and *evm.TU.chr5.993*), encoding endoglucanase (Fig. 8D).

Within the yellow module, a local co-expression network centered on mannan biosynthesis-related genes *evm.TU.chr3.293* (*GbCSLA9A*) and *evm.TU.chr9.476* (*GbCSLA9B*); xylan biosynthesis-related genes *evm.TU.chr11.1607* (putative β-1,4-xylosyltransferase, *IRX14*) and *evm.TU.chr5.417* (putative β-1,4-xylosyltransferase, *IRX9*); and xylan modification-related gene *evm.TU.chr6.1385* (probable glucuronoxylan glucuronosyltransferase, *IRX7*) was identified (Fig. 8E). This network also includes multiple key functional genes, such as xylan modification-related genes *evm.TU.chr1.1515* (glucuronoxylan 4-O-methyltransferase, *GXMT*) and *evm.TU.chr3.1397* (*GXMT*), as well as genes associated with the generation and transport of UDP-sugar precursors, including *evm.TU.chr8.1928* (UDP-glucuronate decarboxylase, *UXS*) and *evm.TU.chr2.1702* (*UXS*), *evm.TU.chr9.7* (UDP-arabinose 4-epimerase, *UXE*), and *evm.TU.chr10.1387* (UDP-rhamnose/UDP-galactose transporter, *URGT*) (Fig. 8E). Moreover, *IRX14* and *IRX9* exhibited strong positive correlations with *GbCSLA9A* and *GbCSLA9B* (Fig. 8F).

In the yellow module, we also identified several transcription factor genes, including *evm.TU.chr1.637* (*GATA9*) and *evm.TU.chr10.213* (*HB15*), which showed significant positive and negative correlations with cellulose and hemicellulose content, respectively (Fig. 8G). Meanwhile, *evm.TU.chr11.910* (*ELF6*) was found to be significantly positively correlated with tracheid length and tracheid width (Fig. 8H and I).

## Discussion

Wood traits are critical determinants of wood properties and application potential. Systematically identifying and characterizing key genes that govern wood traits, and elucidating the molecular mechanisms underlying wood formation, are essential for enhancing wood quality and utilization efficiency. *Ginkgo biloba* wood has excellent material properties and substantial commercial potential. However, compared with conifers and most angiosperms, we still lack a comprehensive understanding of its wood formation mechanisms and the regulatory genes involved in SCW development [11–13]. To address this knowledge gap, we integrated GWAS, TWAS, and WGCNA to identify key genes influencing wood traits in ginkgo and to elucidate the regulatory mechanisms underlying secondary xylem development based on whole-genome resequencing and transcriptome sequencing data from 290 *G. biloba* accessions.

### Genome-wide SNP mutation pattern and transition bias in *G. biloba*

In this study, using whole-genome resequencing data with an average sequencing depth of 7.6× (Table S3), we identified a total of 76 128 881 SNPs and 1 974 424 InDels (Fig. 1A and B), which were subsequently used for GWAS of 12 wood traits in *G. biloba*. Statistical analysis of genome-wide SNP types revealed a transition-to-transversion ratio (TS/TV) of ~1.59 (Fig. 1C), indicating that nucleotide substitutions in this population were predominantly transitions between chemically similar bases (purine to purine or pyrimidine to pyrimidine).

Notably, C:G > T:A transitions exhibited a significantly higher frequency than other mutation types (Fig. 1C), consistent with findings in various species [33, 34]. It has well established that cytosine (C) within CpG dinucleotide contexts is commonly methylated to 5-methylcytosine (5mC), which is prone to spontaneous deamination to thymine (T) [34]. In single-stranded DNA, 5mC is more susceptible to deamination and conversion to thymine, and the resulting thymine does not generate a recognizable G:T mismatch in the absence of a complementary strand, therefore, it is not efficiently detected and repaired by the DNA repair machinery [35, 36]. These molecular mechanisms likely underlie the notable enrichment of C:G > T:A transitions across the genome, providing a plausible biological explanation for the observed mutation distribution pattern in this study.

### Candidate genes related to cellulose synthesis

In the GWAS for cellulose content, we identified a candidate gene, *GbCesA8B* (*evm.TU.chr1.2825*). A frameshift insertion (chr1:997452697) within *GbCesA8B* causing a complete loss of protein function showed a significant association with reduced cellulose content, suggesting that *GbCesA8B* may have a positive effect on cellulose accumulation (Fig. 2D and F). This hypothesis was further supported by the TWAS results, in which the expression level of *GbCesA8B* exhibited a significant positive correlation with cellulose content (Fig. 6F). Notably, among the 93 genes positively associated with cellulose content in the TWAS, three additional *CesA* genes were identified: *GbCesA4* (*evm.TU.chr10.692*), *GbCesA7* (*evm.TU.chr12.1759*), and *GbCesA8A* (*evm.TU.chr1.2824*) (Fig. 6C–E).

We further found that *GbCesA4*, *GbCesA7*, and *GbCesA8A/8B* in *G. biloba* are highly homologous to *AtCesA4*, *AtCesA7*, and *AtCesA8* in *A. thaliana*, respectively (Fig. 2E). Previous studies have demonstrated that cellulose biosynthesis in SCW is catalyzed by the CesA4–CesA7–CesA8 complex, in which each subunit is indispensable [37, 38]. This model has also been validated in *P. trichocarpa*, where deletion of any one of the genes *PtrCesA4*, *PtrCesA7*, and *PtrCesA8* results in prostrate growth and a sharp decrease in cellulose content [2]. Interestingly, *AtCesA4*, *AtCesA7*, and *AtCesA8* in *A. thaliana* exhibit elevated expression specifically in the xylem and display a consistent expression pattern, suggesting that the three CesA subunits are co-expressed in the xylem and form the CesA4–CesA7–CesA8 complex in a fixed proportion [37, 38].

Consistent with this, we observed that *GbCesA4*, *GbCesA7*, *GbCesA8A*, and *GbCesA8B* in ginkgo are not only highly expressed in xylem tissues but also exhibit a strong correlation among their expression levels (Fig. 6G). This suggests that the biosynthesis of SCW cellulose in ginkgo may exhibit a certain degree of evolutionary conservation and may require the synergistic participation of CesA4, CesA7, and CesA8, as observed in angiosperms such as *Arabidopsis* and *Populus*. The results of WGCNA further support this conjecture. The yellow module identified by WGCNA was significantly positively correlated with cellulose content, and its hub genes corresponded exactly to the four *CesA* genes detected in the TWAS, namely *GbCesA4*, *GbCesA7*, *GbCesA8A*, and *GbCesA8B* (Fig. 8D).

These four *CesA* hub genes further formed a co-expression network with several genes closely associated with cellulose biosynthesis, including *TUBA* (*evm.TU.chr2.1590*), *TUBB* (*evm.TU.chr9.972*, *evm.TU.chr10.2089*, and *evm.TU.chr4.2321*), and *EG* (*evm.TU.chr4.1432* and *evm.TU.chr5.993*) (Fig. 8D). *TUBA* and *TUBB* encode α-tubulin and β-tubulin, respectively, which assemble into cortical microtubules that guide the movement of the cellulose synthase complex (CSC) and determine the orientation of cellulose microfibril deposition and cell elongation [39]. *EG* encoded endoglucanase contribute to cellulose degradation and remodeling within the cell wall [40]. Together, we speculate that there may be a potential co-expression mechanism between *CesA* and *TUBA*/*TUBB*, which might help maintain the coordinated interaction between CSC and cortical microtubules to ensure the ordered deposition of cellulose microfibrils. The co-expression of EG within the cellulose synthesis-related gene module indicates that dynamic remodeling may occur concurrently with SCW formation and cellulose biosynthesis. The convergence of statistical association and co-expression network analysis provides strong support for the critical role of these genes in cellulose biosynthesis.

In addition, multiple transcription factors potentially involved in regulating cellulose biosynthesis were identified through TWAS and WGCNA, providing additional insights into the regulatory network underlying SCW formation in ginkgo. The identification of *WRKY12* (*evm.TU.chr7.1702*) suggests that it may play a negative regulatory role in cellulose biosynthesis in ginkgo (Fig. 6H). The function of *AtWRKY12* in *A. thaliana* has been well characterized: loss-of-function mutations in *AtWRKY12* lead to activation of *NST2* expression and ectopic deposition of SCW in pith cells [41], whereas overexpression of *MlWRKY12*, a homolog of *AtWRKY12*, restored the SCW thickening defect in the pith cells of *AtWRKY12* mutants [42]. These findings collectively support the role of *WRKY12* as a suppressor of SCW formation. Consistent with this, our TWAS results revealed a significant negative correlation between *WRKY12* expression and cellulose content in ginkgo, indicating that *WRKY12* may also be involved in the negative regulation of xylem SCW development in ginkgo.

Notably, within the cellulose biosynthesis-related co-expression network in which *GbCesA4*, *GbCesA7*, *GbCesA8A*, and *GbCesA8B* serve as hub genes, we also identified *GATA9* (*evm.TU.chr1.637*) and *HB15* (*evm.TU.chr10.213*), which showed significant positive and negative correlations with cellulose/hemicellulose content, respectively (Fig. 8G). This observation is consistent with previous studies. In *P. trichocarpa*, overexpression of *GATA9* leads to the upregulation of NAC domain transcription factors and downstream SCW biosynthetic genes, thereby promoting SCW thickening in interfascicular fibers [43]; conversely, in *Arabidopsis*, overexpression of *HB15* suppresses SCW formation and disrupts vascular development [44]. Based on these observations, we believe that *GATA9* and *HB15* may play positive and negative roles, respectively, in SCW deposition in ginkgo xylem. However, the exact mechanisms of action and the extent of their influence remain to be clarified through further experimental validation.

As mentioned earlier, *AtCesA4*, *AtCesA7*, and *AtCesA8* encode the cellulose synthase isoforms required for cellulose biosynthesis during SCW formation [37, 38]. In *P. trichocarpa*, five homologous genes, *PtrCesA4*, *PtrCesA7A*, *PtrCesA7B*, *PtrCesA8A*, and *PtrCesA8B*, have been identified as contributors to SCW cellulose deposition [45]. Functional characterization through gene knockout revealed that knockout mutants of *PtrCesA4* and double knockouts of *PtrCesA7a/b* or *PtrCesA8a/b* exhibited a prostrate growth habit and a pronounced reduction in cellulose content, whereas single knockouts of *PtrCesA7a*, *PtrCesA7b*, *PtrCesA8a*, or *PtrCesA8b* did not display obvious growth defects [2]. This observation demonstrates functional redundancy between *PtrCesA7A* and *PtrCesA7B*, as well as *PtrCesA8A* and *PtrCesA8B*, enabling compensation for the loss of one counterpart during cellulose synthesis [2]. In contrast, *PtrCesA4* and *PtrCesA7A/B* and *PtrCesA8A/B* perform similar functions but are not redundant, playing essential roles in cellulose synthesis [2].

Integrating functional annotation with phylogenetic inference, we identified four *CesA* homologous genes in *G. biloba*, namely *GbCesA4*, *GbCesA7*, *GbCesA8A*, and *GbCesA8B*, which are putatively involved in SCW cellulose biosynthesis (Fig. 2E). We also observed that individuals carrying an insertion mutation in *GbCesA8B*, leading to a complete loss of gene function, displayed only a slight reduction in cellulose content without significant growth defects (Fig. 2F). Considering the functional redundancy observed between *PtrCesA8A* and *PtrCesA8B* in *P. trichocarpa* [2], we hypothesize that *GbCesA8A* and *GbCesA8B* in *G. biloba* may also exhibit functional redundancy, and that loss of *GbCesA8B* may potentially compensated by the redundant gene *GbCesA8A*. This hypothesis requires further validation through gene knockout experiments. Notably, no mutations leading to complete loss of function were detected in *GbCesA4* or *GbCesA7*. This observation indicates a potential lack of alternative redundant genes for cellulose synthesis involving *GbCesA4* and *GbCesA7*, thereby subjecting these genes to stronger purifying selection during evolution.

### Candidate genes related to hemicellulose synthesis

In the GWAS for hemicellulose content, we identified a candidate gene, *GbCSLA9B* (*evm.TU.chr9.476*). A non-frameshift deletion (chr9:94004237) within this gene results in the loss of leucine at position 41 and is significantly associated with a decrease in hemicellulose content (Fig. 3D and F). In addition, *GbCSLA9B* exhibits high sequence homology with *AtCSLA9* in *A. thaliana* (Fig. 3E). *AtCSLA9* encodes a β-mannan synthase that localizes to the Golgi membrane [46]. The protein typically comprises an odd number of transmembrane domains, with a catalytic active site oriented toward the Golgi lumen, and catalyzes the synthesis of the backbone of β-1,4-mannan or glucomannan of hemicellulose [46].

Consistently, InterPro domain prediction indicated that GbCSLA9B is also a membrane-bound protein with multiple transmembrane domains, and the leucine deletion at position 41 associated with the decrease in hemicellulose content is located within a transmembrane domain. Therefore, we speculate that *GbCSLA9B* may likewise play an important role in mannan biosynthesis in ginkgo. One possible explanation for the reduced hemicellulose content observed in the deletion mutant is that the loss of amino acid in the transmembrane region may affect the membrane binding ability of GbCSLA9B, thereby limiting its catalytic function in mannan/glucomannan synthesis.

Furthermore, both *GbCSLA9B* and *GbCSLA9A* were found by TWAS to be significantly positively correlated with cellulose content, and both showed high sequence homology to *AtCSLA9*, which encodes a β-mannan synthase in *A. thaliana* (Figs 7A and 3E). This further supports our speculation that *GbCSLA9A* and *GbCSLA9B* are involved in mannan/glucomannan biosynthesis. Notably, *GbCSLA9A* and *GbCSLA9B*, together with *IRX14* (*evm.TU.chr11.1607*) and *IRX9* (*evm.TU.chr5.417*), formed a local co-expression network centered on these genes (Fig. 8E). This network also comprised multiple genes known to be involved in hemicellulose biosynthesis, including *UXS* (*evm.TU.chr2.1702*), *UXE* (*evm.TU.chr9.7*), *URGT* (*evm.TU.chr10.1387*), *IRX7* (*evm.TU.chr6.1385*), and *GXMT* (*evm.TU.chr1.1515* and *evm.TU.chr3.1397*). The yellow module to which this co-expression network belongs is significantly positively correlated with hemicellulose content (Fig. 8A and E).

Within this network, the hub genes *IRX14* and *IRX9* have been reported to catalyze the addition of xylose residues to the xylan backbone via β-1,4-glycosidic linkages through β-1,4-glycosidic linkages [47]. Interestingly, a strong correlation was observed between *GbCSLA9A*/*9B* and *IRX14*/*IRX9* (Fig. 8F). This observation suggests that, despite considerable variation in hemicellulose content within SCW among different ginkgo individuals, the biosynthesis of two major hemicellulose components, mannan and xylan, may exhibit a synergistic relationship, ensuring that the deposition ratio of mannan and xylan remains at a certain range.

Moreover, other genes within this co-expression network are involved in multiple stages of hemicellulose biosynthesis. *UXS* catalyzes the conversion of UDP-glucose to UDP-xylose, providing activated xylose donors for xylan backbone elongation [48]. *UXE* converts UDP-xylose to UDP-arabinose, supplying substrates for arabinosylation of xylan [49]. IRX7 is involved in the formation of the reducing-end tetrasaccharide required for glucuronoxylan/O-acetylglucuronoxylan (GX/AcGX) biosynthesis, a process essential for normal GX/AcGX synthesis and for providing a critical initiation structure for backbone elongation [50]. In *irx7* mutants of *A. thaliana*, AcGX content is reduced by more than 60% [50]. GXMT catalyzes the 4-O-methylation of glucuronic acid substituents of GX, and the degree of 4-O-methylation of GX in GXMT1 mutants is reduced by ~75% compared with the wild type [51]. URGT mediates the transport of UDP-galactose from the cytosol into the Golgi lumen, thereby supporting galactosylation of the mannan backbone [52]. Taken together, this co-expression network centered on *GbCSLA9A*/*9B* and *IRX14*/*9* may be responsible for hemicellulose biosynthesis in ginkgo, encompassing multiple steps from precursor supply and nucleotide sugar transport to polysaccharide polymerization and post-synthetic modification.

We also identified a transcription factor gene, *MYB46* (*evm.TU.chr5.1668*), whose expression level was significantly and positively correlated not only with hemicellulose content but also with *GbCSLA9A*/*9B* (Fig. 7). MYB46 is a secondary master regulator in the NAC-MYB cascade network involved in SCW formation and has been shown to activate a series of structural genes and transcription factors associated with SCW biosynthesis in angiosperms [53]. Notably, previous studies have demonstrated that in *Arabidopsis*, *MYB46* directly binds to the *CSLA9* promoter and drives its transcriptional activity [54]. Accordingly, we speculate that a conserved MYB46–CSLA9 regulatory module may exist and may be involved in mannan synthesis in the xylem of ginkgo.

### Candidate genes related to lignin synthesis

Regarding lignin content, we identified two genes, *evm.TU.chr3.317* and *evm.TU.chr4.1696*, encoding ADT/PDT, both of which were significantly associated with lignin levels (Fig. 5B–C). ADT/PDT is the key rate-limiting enzyme in phenylalanine synthesis, providing initial substrate for the phenylalanine metabolic pathway [55, 56]. ADT/PDT can directly catalyze the dehydration of L-arogenate to phenylalanine, or it can first catalyze the dehydration and decarboxylation of prephenate to phenylpyruvate, which is then converted to phenylalanine through transamination [55, 56]. Since phenylalanine serves as the initial substrate of the phenylpropanoid pathway, its biosynthetic efficiency directly determines the carbon flux through this metabolic route, ultimately influencing the potential accumulation of lignin. Beyond precursor synthesis, several downstream key genes of the phenylpropanoid pathway were identified in TWAS, such as *evm.TU.chr12.1408* (*PAL*) and *evm.TU.chr2.1128* (*PER*), both of which were significantly correlated with lignin content (Fig. 5D–E). PAL, as the first rate-limiting enzyme in this pathway, deaminates phenylalanine to cinnamic acid, thereby providing the substrate for lignin monomer biosynthesis [57]. PER, in contrast, acts at the terminal step, directly catalyzing lignin monomer polymerization and cross-linking, which directly determines lignin deposition of SCW [58]. Importantly, in our results, *evm.TU.chr3.317*, *evm.TU.chr4.1696*, *evm.TU.chr12.1408*, and *evm.TU.chr2.1128* all showed significant positive correlations with lignin content (Fig. 5A–E). These findings indicate that the structural genes identified by TWAS cover multiple key steps in lignin biosynthesis, including the supply of the initial substrate, substrate transformation, and monomer polymerization. Notably, we also identified two *MYB91* genes (*evm.TU.chr8.382* and *evm.TU.chr9.514*) that were positively associated with lignin content (Fig. 5F–G). Previous studies have demonstrated that MYB transcription factors play central roles in regulating lignin biosynthesis by activating diverse structural genes to enhance lignin accumulation [59]. In particular, *MYB91* has been shown to markedly increase lignin levels when transiently overexpressed in pear [60]. This is highly consistent with our findings in ginkgo, suggesting that MYB91 may also be involved in lignin accumulation in ginkgo.

In addition, we identified eight *bHLH* genes that exhibited significant positive correlations with lignin content, including *evm.TU.chr3.1718* (*bHLH6*), *evm.TU.chr3.2340* (*bHLH29*), *evm.TU.chr3.1923* (*bHLH144*), *evm.TU.chr4.1572* (*bHLH80*), *evm.TU.chr9.1343* (*bHLH130*), *evm.TU.chr3.2311*, *evm.TU.chr4.104*, and *evm.TU.chr4.2052* (Fig. 5H). This finding indicates that bHLH transcription factors may function as positive regulators of lignin biosynthesis in ginkgo. Accumulating evidence from other species has established the pivotal roles of multiple bHLH transcription factors in lignin regulation. For instance, in *Populus tomentosa*, *Gossypium hirsutum*, *Brassica campestris*, *Nicotiana benthamiana*, and *Populus alba × Populus glandulosa*, multiple members of the *bHLH* family have been shown to activate the transcription of key genes involved in lignin biosynthesis, including *CCoAOMT*, *PER*, and *POD* [10, 61–63]. Such transcriptional activation ultimately promotes lignin accumulation and deposition, highlighting a conserved regulatory role of bHLH factors in lignin biosynthesis.

Notably, the lignin and flavonoid biosynthetic pathways compete for the common precursor p-coumaroyl-CoA. Chalcone synthase (CHS), the key enzyme in the flavonoid biosynthesis pathway, catalyzes the condensation of p-coumaroyl-CoA and malonyl-CoA to form chalcone, which is the precursor of flavonoids. Because p-coumaroyl-CoA also serves as a key precursor for lignin biosynthesis, CHS activity critically influences the balance between lignin and flavonoid production. Intriguingly, in *Malus domestica*, *MhbHLH130* represses the expression of *MhCHS* by directly binding to its promoter [64], thereby allowing more carbon flux to be directed toward lignin biosynthesis. Consistent with this mechanism, we found that *evm.TU.chr9.1343* (*bHLH130*) shows a significant positive correlation with lignin content in ginkgo. Therefore, we speculate that *bHLH130* in ginkgo may have a positive effect on lignin accumulation, potentially by regulating the metabolic fate of p-coumaroyl-CoA.

### Candidate genes related to wood density and tracheid development

A gene, *evm.TU.chr3.1073* (*TAA1*), encoding L-tryptophan-pyruvate aminotransferase was found in the region associated with WOD (Table S5). TAA1 catalyzes the transfer of amino group from L-tryptophan to pyruvate, resulting in the formation of indole-3-pyruvic acid (IPA), which is a precursor to indole-3-acetic acid (IAA). This pathway is the primary route of IAA biosynthesis in plants. IAA is known to regulate the maintenance and differentiation of cambial cells through ARF transcription factors [65–69]. Therefore, we propose that *evm.TU.chr3.1073* might influence the maintenance or differentiation of cambial cells by affecting IAA synthesis, thereby potentially impacting wood density.

The genes *evm.TU.chr10.1203* (*XYL4*) and *evm.TU.chr7.1369* (*MAN*), associated with WBD, encode xylan 1,4-beta-xylosidase and mannan endo-1,4-beta-mannosidase, respectively (Table S5). *XYL4* and *MAN* play critical roles in the degradation of hemicellulose and the remodeling of SCW structure during wood development [70]. These results suggest that the wood density of ginkgo may be related to SCW remodeling.

Tracheid morphological traits appear to be potentially associated with *evm.TU.chr6.1080* (*EIN3*) and *evm.TU.chr6.1069* (*ABI5*) (Table S5). *EIN3* and *ABI5* encode response factors for ETH and ABA, respectively, playing pivotal roles in the corresponding signal transduction pathways. ABA and ETH have been found to regulate cell elongation and SCW synthesis by promoting H_2_O_2_ production in *Arabidopsis* and cotton fibers [71–73]. During the early stage of fiber cell development in *Gossypium hirsutum*, a low concentration of H_2_O_2_ facilitates cell elongation [74]. However, at the transition stage, ABA and ETH may inhibit fiber elongation and promote SCW synthesis by modulating high levels of H_2_O_2_ and calcium ions [74]. Hence, we postulate that *EIN3* and *ABI5* might influence the development of tracheid cells in ginkgo xylem through ETH and ABA signal transduction.

In addition, we identified *evm.TU.chr11.910* (*ELF6*) in the yellow module related to cellulose/hemicellulose synthesis, and its expression was significantly positively correlated with tracheid length and width (Fig. 8H–I). Previous studies have shown that ELF6 relieves the epigenetic silencing effect on expansion genes in flower organs of *Arabidopsis*, thereby promoting elongation of carpel cells [75]. Based on our results, it is speculated that ELF6 may affect tracheid morphology in ginkgo xylem through a similar mechanism.

### Limitation

In TWAS and WGCNA, genes with FPKM <0.5 were removed to reduce noise and improve the reliability of the results. However, this filtering strategy may have excluded some lowly expressed genes that nonetheless play important roles in SCW development. In addition, this study primarily identified candidate genes and transcription factors associated with wood traits and SCW development through GWAS, TWAS, and WGCNA, but lacks functional validation. Future studies should employ gene-editing approaches or transgenic techniques to functionally validate these key genes and further clarify their specific roles in wood trait formation.

## Conclusion

In this study, we integrated GWAS, TWAS, and WGCNA to elucidate the genetic basis and regulatory mechanisms underlying wood trait variation and SCW development in *G. biloba*. Multiple lines of evidence, including significant GWAS signals, expression–trait associations, co-expression networks, and phylogenetic analyses, consistently support the synergistic core role of *GbCesA4*, *GbCesA7*, *GbCesA8A*, and *GbCesA8B* in the cellulose biosynthesis of SCW in ginkgo. The co-expression of these *CesA* genes with microtubule-related genes (*TUBA* and *TUBB*) and endoglucanase genes (*EG*) indicates a tight coordination between cellulose biosynthesis, cytoskeletal guidance, and dynamic cell wall remodeling. Moreover, the mild phenotypic decline of loss-of-function mutations of *GbCesA8B* implies that *GbCesA8A* and *GbCesA8B* in ginkgo may have functional redundancy, similar to *PtrCesA8A* and *PtrCesA8B* in *P. trichocarpa*, whereas the absence of loss-of-function mutations in *GbCesA4* and *GbCesA7* might be due to the lack of redundant genes, subjecting these genes to stronger purifying selection. In addition to structural genes, TWAS and WGCNA identified several transcription factors potentially involved in the regulation of SCW development, among which *GATA9* may act as a positive regulator, whereas *WRKY12* and *HB15* may function as negative regulators of SCW formation.

For hemicellulose biosynthesis, we identified *GbCSLA9A*/*9B* as key contributors to mannan/glucomannan synthesis, forming a tightly connected co-expression network with xylan biosynthetic genes, including *IRX9* and *IRX14*. This network encompasses genes such as *UXS*, *UXE*, *IRX7*, *GXMT*, and *URGT*, which are involved in multiple stages of hemicellulose biosynthesis, ranging from the supply and transport of nucleotide sugars to the elongation and modification of polymers. This indicates a coordinated mechanism that maintains a balanced deposition of major hemicellulose components in the SCW. In addition, the significant association of *MYB46* with both hemicellulose content and *CSLA9* further supports the possible existence of a *MYB46*–*CSLA9* module regulating mannan synthesis in ginkgo.

The key genes involved in phenylalanine biosynthesis (*ADT*/*PDT*), phenylalanine metabolism (*PAL*), and lignin polymerization (*PER*) are all significantly correlated with lignin content, indicating that lignin biosynthesis in ginkgo appears to be regulated through both precursor supply and downstream polymerization processes. In addition, multiple transcription factors, including *MYB91* and several members of the *bHLH* family, are believed to positively influence on lignin accumulation, among which *bHLH130* may affect lignin accumulation by modulating the carbon flux allocation between lignin and competing metabolic pathways.

## Materials and methods

### Plant materials and wood properties determination

A total of 290 ginkgo accessions were collected in August 2023 from major ginkgo distribution regions in China. Detailed sampling information is provided in Table S1. For each tree, two to three young leaves were collected for whole-genome resequencing, and secondary xylem tissue was obtained after removing bark for transcriptome sequencing. Xylem materials were drilled using a growth cone with an inner diameter of 5.0 mm for the determination of wood properties.

Wood basic density (WBD) and wood oven-dry density (WOD) were determined using the drainage method. The wood cores were soaked in deionized water until fully saturated, and their saturated volume was measured by the water displacement method and recorded as V_1_ (cm^3^). The cores were then oven-dried at 60°C to constant mass, the oven-dry mass was determined and recorded as m (g), and the oven-dry volume was measured by the water displacement method and recorded as V_2_ (cm^3^). The calculation formulas for WBD and WOD are as follows:


$$ \mathrm{WBD}\ \left(\mathrm{g}\cdotp{\mathrm{cm}}^{-3}\right)=\mathrm{m}/{\mathrm{V}}_1. $$

$$ \mathrm{WOD}\ \left(\mathrm{g}\cdotp{\mathrm{cm}}^{-3}\right)=\mathrm{m}/{\mathrm{V}}_2. $$

Tracheid morphological characteristics, including tracheid length (TL), tracheid width (TW), tracheid wall thickness (TWT), tracheid length to width ratio (TLW), and tracheid width to lumen ratio (TWL), were measured using the Franklin macerating method [76]. Wood chips were macerated by soaking in mixed solution (1:1) of glacial acetic acid and 30% hydrogen peroxide in a 90°C water bath until the chips turned completely white. After cooling, they were rinsed with deionized water. The tracheid suspension was pipetted onto a glass slide (one to two drops) and stained with 1% safranin solution. Images were acquired using a DM7500 binocular microscope (Leica, Wetzlar, Germany) equipped with an ICC50W camera system (Leica, Germany). TL (μm), TW (μm), and tracheid lumen diameter (TLD, μm) were measured using LAS EZ software. The calculation formulas for TWT, TLW, and TWL are as follows:


$$ \mathrm{TWT}\ \left(\mathrm{\mu} \mathrm{m}\right)=\left(\mathrm{TW}-\mathrm{TLD}\right)/2. $$

$$ \mathrm{TLW}=\mathrm{TL}/\mathrm{TW}. $$

$$ \mathrm{TWL}=\mathrm{TW}/\mathrm{TLD}. $$

Microfibril angle (MFA) was calculated by 0.6 T method based on diffraction pattern of the cross-section of the wood core [77]. The diffraction pattern of the wood core was scanned using an X-ray diffractometer (Ultima IV, Rigaku, Tokyo, Japan) with parameters as 40 kV tube voltage, 30 mA current, 90° - 270° scanning range, 72°/min scanning speed, and 0.36° step size. The MFA (°) was calculated by 0.6 T method using Origin 2025 software.

Cellulose crystallinity degree (CCD) was calculated by Segal empirical method based on diffraction pattern of wood powder [78]. The diffraction pattern of the wood powder was scanned using an X-ray diffractometer with parameters as 40 kV tube voltage, 30 mA tube current, 5° - 40° scanning range, and 6°/min scanning speed. The calculation formula for CCD is as follow:


$$ \mathrm{CCD}\ \left(\%\right)=\left(\mathrm{Iu}-\mathrm{Ia}\right)/\mathrm{Ia}. $$

In the formula, Iu is the maximum intensity at 2θ = 22°, and Ia is the minimum intensity at 2θ = 18°.

Lignin content (LC) was measured using kits produced by Beijing Boxbio Science & Technology Co., Ltd. Cellulose content (CC) and hemicellulose content (HCC) was measured using kits produced by Beijing Solarbio Science & Technology Co., Ltd. Phenotypic data for all wood properties are presented in Table S2.

### Whole-genome resequencing and variant detection

Whole-genome resequencing was performed by Beijing Novogene Co, Ltd. DNA was extracted from the samples using the cetyltrimethylammonium bromide (CTAB) method. After passing the quality assessment, DNA samples were randomly sheared into ~350 bp fragments using an ultrasonic disintegrator. Subsequently, the DNA fragments were subjected to end repair, poly(A) tailing, adaptor ligation, fragment selection, PCR amplification, and purification. Finally, library construction was completed using TruSeq Library Construction Kit (Illumina, California, USA). After the library passed quality control, sequencing was performed on the DNBSEQ-T7 platform. Quality control of raw data was performed to remove paired reads with adapters, paired reads with more than 10% N (unknown base), and paired reads with more than 50% low quality bases (Q ≤ 5).

The clean reads were aligned to the *Ginkgo* reference genome [79] using the BWA software [80], and the alignment results were deduplicated using SAMtools [81]. Variant detection was performed using the HaplotypeCaller module in GATK 4.5 software [82], and the VCF files were merged using CombineGVCFs. Low-quality variant sites were initially filtered using the vcfutils.pl script in the SAMtools tool, with the parameters -w 5 -W 2. Single nucleotide polymorphisms (SNPs) and insertion–deletion variants (InDel) were extracted using the SelectVariants module in GATK, and further hard-filtered using the VariantFiltration module of GATK software. Among them, the hard-filter criteria of SNP were QD < 2.0||MQ < 40.0||FS > 60.0||SOR > 3.0||MQRankSum < −12.5||ReadPosRankSum < 8.0, and the hard-filter criteria of InDel were QD < 2.0||FS > 200.0||SOR > 10.0||ReadPosRankSum < −20.0. Finally, vcftools software was used to filter again with --min-mean DP 5 --maf 0.05 --max-missing 0.95 --minQ 30 --minGQ 0 --min-alleles 2 --max-alleles 2 as criteria to obtain final high-quality SNPs and InDels. SNPs and InDels were functionally annotated using the table_annovar.pl script in ANNOVAR software [83].

### Transcriptome sequencing and expression quantification

Transcriptome sequencing was conducted by Beijing Novogene Co, Ltd. Total RNA was extracted using the RNAprep Pure Plant Plus Kit (Tiangen Biotech, Beijing, China). After enrichment of mRNA, fragmentation was performed using Fragmentation Buffer to generate short fragments, which were then reverse transcribed into double-stranded cDNA. The resulting cDNA was purified with AMPure XP beads (Beckman Coulter, California, USA), followed by end repair, addition of an adenine tail, and adaptor ligation. Size selection of cDNA fragments (~370–420 bp) was carried out using AMPure XP beads. Subsequently, PCR amplification was performed, and the PCR products were purified with AMPure XP beads to construct the sequencing libraries. The integrity and insert size of the fragments of the libraries were evaluated using a Fragment Analyzer (Agilent Technologies, California, USA), while the effective concentration of the library was detected by qRT-PCR. Qualified libraries were sequenced on the Illumina NovaSeq 6000 platform (Illumina, California, USA). Raw reads obtained from sequencing were subjected to quality control to remove paired reads with adapters, paired reads with more than 10% N (unknown base), and paired reads with more than 50% low quality bases (Q ≤ 5). Clean reads were then aligned to the reference genome using HISAT2 [84]. Gene expression levels were quantified by counting the number of reads mapped to each gene using HTSeq [85], and normalized as FPKM values in R.

### Genome-wide association study

GWAS were conducted using the linear mixed model (LMM) of GEMMA and EMMAX, both of which considered population structure and kinship as covariates. Linkage disequilibrium (LD) filtration of SNPs and InDels were performed using plink with the parameters --indep-pairwise 50 10 0.2. After filtering, 7 207 684 independent SNPs and 302 430 independent InDels were retained. Accordingly, the significance thresholds of GWAS were set to 1.39 × 10^−7^ for SNPs and 3.31 × 10^−6^ for InDels, based on the Bonferroni correction (1/*n*). Manhattan plots were used to visualize the genome-wide distribution of *p*-values for SNPs and InDels, while quantile-quantile (QQ) plots were used to compare the observed *p*-value distribution with the theoretical expectation. For loci exceeding the significance threshold, genomic regions spanning 100 kb upstream and downstream of each significant SNP or InDel were defined as associated regions. Genes overlapping more than 10% with these associated regions were further screened. Based on functional annotation of SNPs and InDels, we retained genes containing non-synonymous SNPs or frameshift/non-frameshift InDels, or containing SNPs/InDels within the upstream promoter region. In addition, further reanalysis was conducted in combination with the transcriptome data of xylem tissues at different developmental stages in my published literature [16]. Genes with low expression (mean FPKM < 0.5) were excluded, and the remaining genes were considered as the final set of candidate genes.

### Transcriptome-wide association study

After filtering out low-expression genes (mean FPKM < 0.5) and retaining 18 203 genes, transcriptome-wide association study was performed using the linear mixed model (LMM) of GEMMA software based on phenotypic data of wood traits and gene expression data. Before association, the kinship matrix calculated by GEMMA and incorporated as a covariate into the association analysis. The significance threshold calculated as FDR × *n*/*m*, where FDR = 0.01, n represents the number of genes with *P*-values <.01, and m is the total number of genes involved in TWAS [86]. The significance thresholds in TWAS of WBD, WOD, MFA, CCD, TL, TW, TWT, TLW, TWL, LC, CC, and HCC were 1.12 × 10^−4^, 1.92 × 10^−4^, 5.49 × 10^−5^, 5.16 × 10^−3^, 1.19 × 10^−3^, 4.34 × 10^−3^, 2.22 × 10^−3^, 1.38 × 10^−4^, 1.23 × 10^−4^, 1.84 × 10^−3^, 9.94 × 10^−4^, and 7.84 × 10^−4^, respectively. Manhattan plots were used to visualize the distribution of *p*-values of genes in each chromosome.

### Weighted gene co-expression network analysis

Weighted gene co-expression network analysis was performed using the WGCNA R package. Genes with low expression (mean FPKM < 0.5) were removed prior to network construction. Samples were hierarchically clustered with the hclust function in R, and outliers were excluded. The correlation between genes was calculated through the weighted correlation coefficient, and the adjacency matrix was calculated. The pickSoftThreshold function was applied to calculate the fitting degree and connectivity of the scale-free network under different soft thresholds, and the optimal soft threshold was automatically selected to satisfy the scale-free network. A topological overlap matrix (TOM) was then calculated, and genes were hierarchically clustered based on TOM. Modules were identified by dynamically cut the gene clustering tree and set the minimum number of module genes to 30. Modules with highly correlated eigengenes were subsequently merged using a merge cut height of 0.235. Correlations between module eigengenes and wood traits, along with *P*-values, were calculated, and a module-trait correlation heatmap was generated. Key modules were exported to Cytoscape [87] for network visualization.

## Supplementary Material

Web_Material_uhag062

## Data Availability

The raw sequencing data of whole-genome resequencing and transcriptome sequencing were uploaded to the National Genomics Data Center (NGDC, https://ngdc.cncb.ac.cn/) with the accession code PRJCA047587 and PRJCA048147.
